# Insomnia as a risk factor for the development of depression and anxiety in primary care: a matched population-based cohort study

**DOI:** 10.1093/fampra/cmag019

**Published:** 2026-04-20

**Authors:** Maria C N Meijer, Rutger A Middelburg, Ingrid A Arnold, Jeanine Kamphuis, Dennis O Mook-Kanamori, Mattijs E Numans, Niels H Chavannes, Robert A Schoevers, Jens H van Dalfsen

**Affiliations:** Department of Public Health and Primary Care, Leiden University Medical Centre (LUMC), Albinusdreef 2, 2333ZA Leiden, The Netherlands; Department of Public Health and Primary Care, Leiden University Medical Centre (LUMC), Albinusdreef 2, 2333ZA Leiden, The Netherlands; Department of Public Health and Primary Care, Leiden University Medical Centre (LUMC), Albinusdreef 2, 2333ZA Leiden, The Netherlands; Department of Psychiatry, University Medical Centre Groningen (UMCG), Hanzeplein 1, 9713GZ Groningen, The Netherlands; Department of Public Health and Primary Care, Leiden University Medical Centre (LUMC), Albinusdreef 2, 2333ZA Leiden, The Netherlands; Department of Public Health and Primary Care, Leiden University Medical Centre (LUMC), Albinusdreef 2, 2333ZA Leiden, The Netherlands; Department of Public Health and Primary Care, Leiden University Medical Centre (LUMC), Albinusdreef 2, 2333ZA Leiden, The Netherlands; Department of Psychiatry, University Medical Centre Groningen (UMCG), Hanzeplein 1, 9713GZ Groningen, The Netherlands; Department of Psychiatry, University Medical Centre Groningen (UMCG), Hanzeplein 1, 9713GZ Groningen, The Netherlands

**Keywords:** sleep initiation and maintenance disorders, insomnia, depression, depressive disorder, anxiety, anxiety disorder, general practice, primary health care

## Abstract

**Background:**

While insomnia, depression and anxiety disorders are most frequently diagnosed and treated in primary care, the longitudinal relationship between these phenomena remains elusive in this setting. The present study aimed to quantify the association of registered insomnia with subsequent depression or anxiety in primary care.

**Methods:**

A matched population-based cohort study was conducted using International Classification of Primary Care (ICPC) and Anatomical Therapeutic Classification (ATC) codes extracted from medical records of 84 Dutch general practices. Patients without documented psychological or social problems, aged 18 to 65 years, with a registered insomnia diagnosis (ICPC: P06) or prescribed sleep medication (ATC: N05CD, N05CF, N05CH) were matched 1:1 to healthy controls on sex, year of birth, and general practice. Controls could be used more than once. The primary outcome measure was depression (ICPC: P03, P76, P76.02) or anxiety (ICPC: P01, P74, P74.01, P74.02) registration up to five years after the insomnia diagnosis.

**Results:**

Patients with insomnia between 1 January 2010 and 1 January 2015 (n = 1535) and the control group (n = 1502) were followed for five years after the insomnia diagnosis. Patients with insomnia had a higher risk of depression or anxiety (odds ratio (OR) = 1.53, 95% confidence interval (CI) [1.18–1.98]).

**Conclusions:**

Patients with an insomnia diagnosis in primary care were at higher risk to develop depression or anxiety compared to healthy controls. This finding identifies insomnia as a potential marker for early detection and prevention of depression and anxiety in primary care.

Key messages

- Insomnia represents a well-established risk factor for the development of depression and anxiety, although the clinical implications of this relationship for primary care remain elusive.
- Identifying insomnia as a risk factor for depression and anxiety in primary care could improve vigilance and prevention in patients at risk for depression and anxiety.
- This matched population-based cohort study demonstrated that patients with insomnia in primary care have a 1.5-fold increased risk of developing depression or anxiety, despite provision of usual care.
- The higher risk for depression and anxiety in patients with insomnia illustrates the need for adequate insomnia treatment in primary care, including an enhanced implementation of cognitive behavioural therapy for insomnia (CBT-I) as a promising prevention strategy for mental disorders.

## Introduction

Depressive and anxiety disorders are the most prevalent mental disorders in the general population, affecting 279.6 and 301.4 million individuals worldwide. Between 1990 and 2019, the global burden of both conditions increased rapidly, and these numbers are expected to continue to rise [1]. Of all mental disorders, depressive and anxiety disorders account for the largest proportion of disability-adjusted life years (DALYs) lost and have the largest share in economic burden [1, 2]. The identification of potential targets for the prevention of depression and anxiety disorders is therefore essential.

General practitioners (GPs) have a pivotal role in the prevention, diagnosis, and treatment of depression and anxiety disorders. The majority of patients with depression and anxiety disorders receive the diagnosis and treatment from their GP [3, 4]. Each year, 11 of 1000 patients registered in primary care receive a diagnostic code for depressive disorder in the electronic medical record (EMR), and another 11 per 1000 receive a code for anxiety disorder. The symptom codes “down or depressive feeling” and “anxious, nervous, or tense feeling” are entered in the medical record at rates of 11 and 14 per 1000 patients per year [5]. Despite the fact that diagnostic criteria for depression or anxiety disorders are met in nearly all cases [6, 7], GPs often prefer to record the symptoms rather than the disorders [8, 9]. Therefore, GPs see considerably more patients with depression and anxiety disorders than are formally diagnosed and documented.

GPs are frequently visited for a broad range of mental, physical, and social conditions, providing opportunities for identifying patients at risk for depression and anxiety and patients with subclinical symptoms. Implementing interventions in patients at risk (selective prevention) and in patients with subclinical symptoms (indicated prevention) [10] requires the identification of modifiable patient characteristics associated with the development of depression and anxiety.

Evidence suggests that insomnia could help with the identification of individuals at increased risk for depression and anxiety. Insomnia is a sleep disorder characterized by difficulties initiating sleep or maintaining sleep, or early morning awakening, leading to daytime dysfunction. It has a close relationship with depression and anxiety [11]. Besides a core symptom of most depressive disorders and generalized anxiety disorder [12], insomnia is a prodromal symptom that frequently precedes depression and anxiety onset [13, 14], Recent meta-analyses of longitudinal studies further showed that individuals with insomnia have a two-to-three-fold higher risk of developing a depressive or anxiety disorder, relative to healthy controls [15, 16].

Insomnia is inadequately managed in primary care, with three-quarters of Dutch patients receiving sleep medication despite its long-term ineffectiveness and side effects [17]. Insomnia can be effectively and safely treated with Cognitive Behavioural Therapy for Insomnia (CBT-I), with more durable effects than sleep medication [18]. For this reason, CBT-I is the recommended first-line treatment in clinical guidelines [19]. Recent studies highlight the potential of CBT-I to prevent mental disorders and to reduce their severity in patients with comorbid insomnia [11, 20]. Therefore, establishing a relationship between insomnia and the development of depression and anxiety in the primary care setting could establish a promising target for prevention. The present study aimed to quantify the association between an insomnia diagnosis in primary care and subsequent depression and anxiety in a five-year follow-up period.

## Methods

### Study design and study population

A matched population-based cohort study was conducted, utilising routine healthcare data from 84 general practices in the South-West of the Netherlands. Patients aged 18 to 65 years, registered at an ELAN (Extramural LUMC Academic Network) general practice between 1 January 2010 and 1 January 2015, with a minimal registration duration of five years, were eligible for inclusion. GP registration was assessed as having a quarterly registration fee, as GPs in the Netherlands receive this fee every three months for each registered patient. Patients with missing data for sex, year of birth, or medical history were excluded. All patients with a first insomnia diagnosis or insomnia medication prescription code between 1 January 2010 and 1 January 2015 were included in the insomnia group (see: Insomnia). All patients with documented psychological problems (ICPC P codes), social problems (ICPC Z codes), registered use of psychoactive medication (ATC N05 and N06 codes) or anorexia or bulimia nervosa (ICPC code T06) on or before the index date were excluded to reduce the effect of reverse causality. A control group of patients without a documented diagnosis, or prescription for insomnia, or documented psychological or social problems, registered use of psychoactive medication, or anorexia or bulimia nervosa, was matched on sex, year of birth, and general practice in a 1:1 ratio [21]. Matching was performed with replacement. Therefore, control patients could be matched to more than one patient with insomnia. The index dates of both the patient with insomnia and the matched control were set to the insomnia diagnosis date. Patients with fewer than five years of follow-up after the index date were excluded. If a matched control patient had fewer than five years of follow-up, the control patient was replaced by a new control patient with five years of follow-up after the index date.

### Data

Patient data from ELAN were used for this study [22]. This database contains pseudonymized EMR data from approximately 570 000 patients registered at 84 primary care facilities in the South-West of the Netherlands. The original data was extracted from the EMR and pseudonymized by a trusted third party. The database includes demographic information (year of birth, sex), consultation dates, diagnosis and symptom codes, according to the ICPC-1 of the World Health Organisation (WHO) [23], and prescribed medication registered in ATC codes [24].

### Insomnia

Patients with insomnia were identified based on either the ICPC code for insomnia (P06) or ATC codes indicating hypnotic prescriptions of benzodiazepine derivatives (N05CD), hypnotic benzodiazepine related drugs (N05CF), and melatonin receptor agonists (N05CH). Patients with sleep apnoea (ICPC P06.01) or in whom the P06 code description contained “apnoea” were excluded.

### Depression and anxiety

Since GPs often record depression and anxiety as “symptoms” [8, 9], depression and anxiety symptom codes were included in the definitions of depression and anxiety. Depression was defined as an ICPC symptom code for “down or depressive feeling” (P03) or ICPC diagnosis code for depressive disorder (P76), including the ICPC code for dysthymia (P76.02). Postpartum depression (P76.01) was excluded. Anxiety was defined as an ICPC symptom code for “anxiety, nervous, or tense feeling” (P01) or ICPC diagnosis code for anxiety disorder or state of anxiety (P74), panic attacks or disorder (P74.01), or generalized anxiety disorder (P74.02). Depression or anxiety was defined as the presence of any of the codes included in either of the two above definitions.

### Somatic comorbidity

ICPC codes with integers under 60 represent symptoms, complaints, and actions, whereas codes with integers above 60 represent diagnoses and conditions [23]. For example, T01 represents “excessive thirst”, and T90 represents “Diabetes mellitus”. Per patient, the number of distinct ICPC codes above 60 was examined to assess the degree of somatic comorbidity at the index date.

### Statistical analysis

Statistical analyses were performed using SPSS (SPSS 29.0 statistics for Windows, IBM, Armonk, New York). Baseline characteristics were summarized using descriptive statistics, reporting numbers (*n*), percentages (%), medians, and interquartile ranges (IQR) for both groups. Numbers of cases with depression and anxiety in the insomnia and the control groups, during the five-year follow-up period, were reported in absolute numbers (*n*) and in percentages (%), as were symptoms and disorders separately for both conditions. Univariate logistic regression analyses were performed to quantify the odds ratio for depression or anxiety in the insomnia group versus the control group (Model 1). Multivariate logistic regression analyses were performed to adjust this odds ratio for comorbidity using the number of distinct ICPC codes above 60 (Model 2). To minimize the potential influence of reverse causality, these analyses were repeated for depression or anxiety occurring six months or more after the index date (Model 3). If multiple diagnoses or symptom codes were present during follow-up, the first symptom or diagnosis code date was used. All analyses were performed for depression, anxiety, and depression or anxiety combined. For all logistic regressions, results were reported as odds ratios (OR) with 95% confidence intervals (CI).

As opposed to lumping symptoms and diagnosed disorders together as one, sensitivity analyses were performed to make a distinction between components of the definitions of depression and anxiety. All logistic regression analyses were repeated for the outcomes of depressive symptoms (P03), depressive disorder (P76, P76.02), anxiety symptoms (P01), and anxiety disorder (P74, P74.01, P74.02) separately.

To account for double selection of control patients, based on matching with replacement, doubly selected control patients were weighted as 0.5 in all analyses.

## Results

From the 371 870 patients in the ELAN database, aged between 18 and 65, between 1 January 2010 and 1 January 2020, 233,306 patients were excluded because there was no documented registration fee (*n* = 2864) or the period during which a registration fee was documented was shorter than five years (*n* = 230 442). Of the remaining 138 564 patients, 14 883 patients were selected, based on ICPC code P06 (*n* = 4967), ATC codes N05CD, N05CF, N05CH (*n* = 13 359), or a combination of these codes. Of these, 1742 patients were excluded because follow-up from the index date (i.e. date of ICPC or ATC code for insomnia) was shorter than five years (*n* = 1209) or age at index date was under 18 or above 65 years (*n* = 533). From the remaining 13 141 patients, 11 075 patients with a prior ICPC P (*n* = 8136), Z (*n* = 3435), T06 (*n* = 88) code, an ATC N05 or N06 prescription code (*n* = 9352), or a combination of these codes before the index date were excluded.

**Figure 1:**
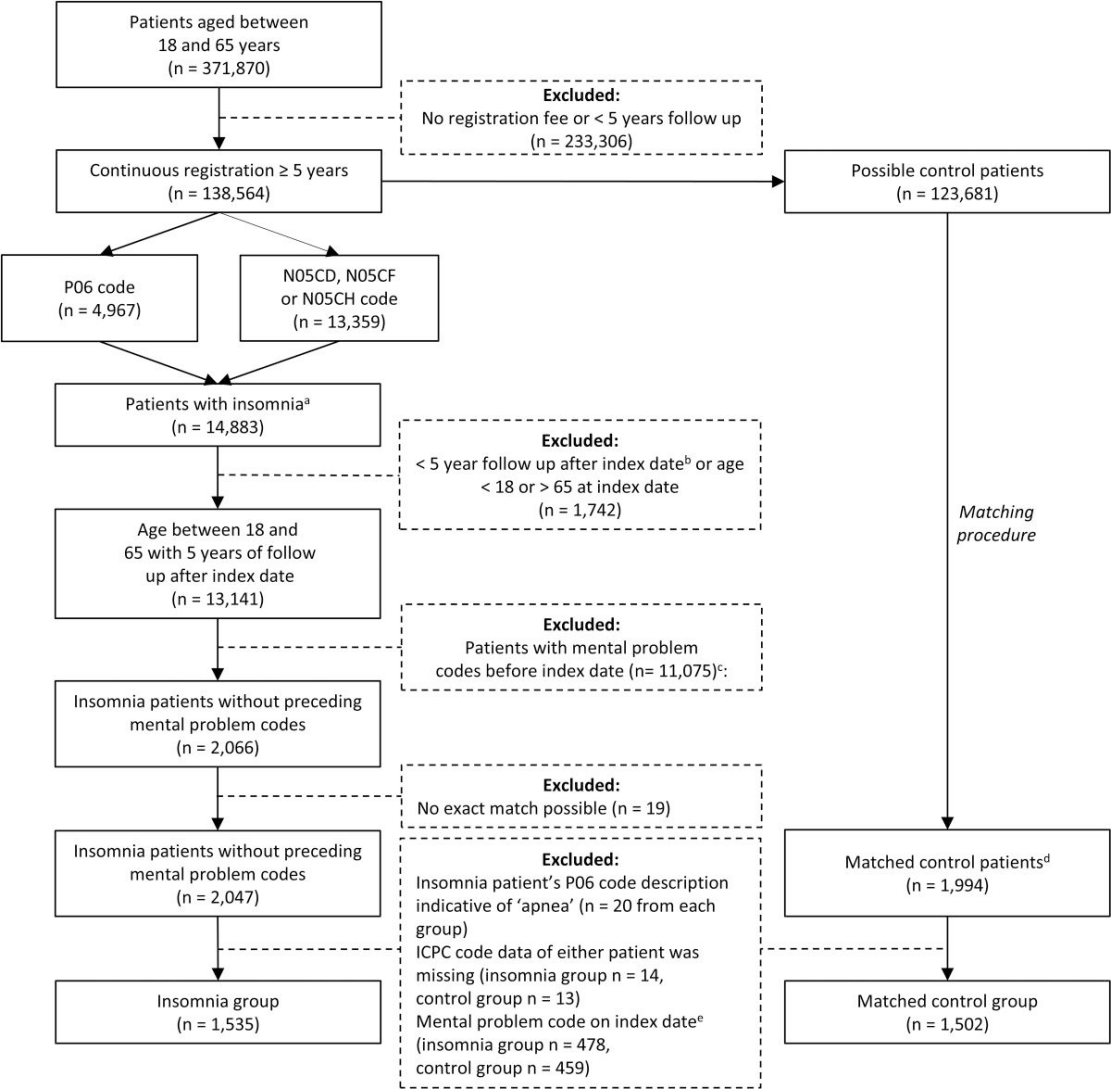
Flowchart of data collection. All included patients were between 18 and 65 years old between 1 January 2010 and 1 January 2015, and were followed for 5 years after the index date. ^a^Some patients had both a P06 code and a N05CD, N05CF or N05CH code. ^b^Index date is marked as the first diagnosis of insomnia, either through P06, N05CD, N05CF or N05CH code present between 1 January 2010 and 1 January 2015. ^c^Reasons for exclusion: P codes (*n* = 8136) Z codes (*n* = 3435) T06 (*n* = 88) N05 or N06 (*n* = 9352). P codes include all psychiatric symptoms as well as psychiatric diagnoses, Z codes include social problems, T06 represents anorexia nervosa and bulimia nervosa, and N05 and N06 prescriptions together represent all psychoactive medications. Most patients had more than one reason for exclusion. ^d^Some control patients that were matched had P, Z, T06, N05 or N06-codes before the index date. These control subjects were excluded, and new controls without previous mental problem codes were matched to the insomnia patient. ^e^Matches in which one of the patients had had a P, Z, or T06 diagnoses codes or N05 or N06 codes on the index date were excluded. Reasons for exclusion: P, Z, T06 codes (*n* = 386 in the insomnia group, *n* = 370 in the control group), N05 or N06 (*n* = 92 in the insomnia group, *n* = 91 in the control group).

The remaining 2066 patients with insomnia were matched on year of birth, sex, and general practice to 1994 control patients without an ICPC P06, ATC N05CD, N05CF, or N05CH code on index date, and without ICPC P, Z, T06, and ATC N05 or N06 codes before the index date. Nineteen insomnia patients had no exact match and were therefore excluded. Following the matching procedure, several matches (i.e. the patient in the insomnia group and the matched control patient) were excluded, because the P06 code description of insomnia patients contained sleep apnoea (*n* = 20 in each group), diagnosis code data was missing of one of the patients (*n* = 14 in the insomnia group, *n* = 13 in the control group), or one of the patients had a P, Z, T06 code (*n* = 386 in the insomnia group, *n* = 370 in the control group), or N05 or N06 medication prescriptions (*n* = 92 in the insomnia group, *n* = 91 in the control group) on the index date, other than P06, N05CD, N05CF, or N05CH in the insomnia group. The final study population consisted of 1535 and 1502 patients in the insomnia and control groups, with 33 control patients matched to two insomnia patients (Fig. 1).

The insomnia group consisted of 667 (43.5%) male and 868 (56.5%) female patients, the control group of 660 (43.9%) male and 842 (56.1%) female patients. The median age in the insomnia group was 44.28 (IQR = 23.11) and in the control group, 44.45 years (IQR = 22.91). The median number of ICPC codes above 60 was 5 (IQR = 5) in the insomnia group and 4 (IQR = 5) in the control group (Table 1).

**Table 1:** Baseline characteristics.

	Insomnia	Control	Total
Sex	*n* (%)	*n* (%)	*n* (%)
Male	667 (43.5)	660 (43.9)	1327 (43.7)
Female	868 (56.5)	842 (56.1)	1710 (56.3)
Age	*Median (IQR)*	*Median (IQR)*	*Median (IQR)*
Years	44.28 (23.11)	44.45 (22.91)	44.40 (22.98)
Comorbidity	*Median* (IQR)	*Median* (IQR)	*Median* (IQR)
ICPC codes above 60	5 (5)	4 (5)	4 (5)

Note 1. ICPC, International Classification of Primary Care.

Note 2. IQR, interquartile range.

Note 3. Due to matching with replacement, 33 double-used control patients were weighted as 0.5, all other patients were given a weight of 1.0 in the analyses.

In the insomnia and control groups, 206 (13.4%) and 117 (7.8%) patients developed depression or anxiety. Insomnia was associated with the development of depression or anxiety in Model 1 (OR = 1.84, 95% CI [1.45–2.34]), in Model 2 (OR = 1.81, 95% CI [1.42–2.30]) and in Model 3 (OR = 1.53, 95% CI [1.18–1.98]). A supplementary analysis including only anxiety and depression cases occurring from 12 months after the index date (adjusted for somatic comorbidity) showed an OR of 1.50 (95% CI [1.14–1.97]) for the insomnia group relative to the control group. Insomnia was associated with the development of depression in Model 3 (OR = 2.03, 95% CI [1.39–2.97]). The OR for development of anxiety in Model 3 was 1.21 (95% CI [0.89–1.66]).

Sensitivity analyses showed that insomnia was associated with subsequent depressive disorder (OR = 2.84, 95% CI [1.71–4.71]) in Model 3. The OR for the development of depressive symptoms was 1.39 (95% CI [0.78–2.45]), for anxiety disorder 1.01 (95% CI [0.59–1.73]) and for anxiety symptoms 1.39 (95% CI [0.95–2.02]) in Model 3 (Table 2).

**Table 2:** Risk of depression and anxiety in the insomnia versus the control group^a^.

	Insomnia^b^	Control^b^	OR [95% CI]		
	*n* (%)	*n* (%)	Model 1^c^	Model 2^c^	Model 3^c^
Depression^d^	109 (7.1)	45 (3.0)	2.48 [1.74—3.53]	2.52 [1.77—3.61]	2.03 [1.39—2.97]
Disorder^e^	78 (5.1)	21 (1.4)	3.78 [2.32—6.15]	3.78 [2.31—6.16]	2.84 [1.71—4.71]
Symptoms^f^	36 (2.3)	24 (1.6)	1.48 [0.88—2.49]	1.53 [0.90—2.58]	1.39 [0.78—2.45]
No depression^d^	1426 (92.9)	1457 (97.0)	reference	reference	reference
Anxiety^g^	111 (7.2)	83 (5.5)	1.34 [1.00—1.80]	1.29 [0.96—1.73]	1.21 [0.89—1.66]
Disorder^e^	34 (2.2)	32 (2.1)	1.04 [0.64—1.70]	1.01 [0.62—1.65]	1.01 [0.59—1.73]
Symptoms^f^	81 (5.3)	51 (3.4)	1.60 [1.12—2.29]	1.54 [1.07—2.22]	1.39 [0.95—2.02]
No anxiety^g^	1424 (92.8)	1420 (94.5)	reference	reference	reference
Depression or anxiety^d,g^	206 (13.4)	117 (7.8)	1.84 [1.45—2.34]	1.81 [1.42—2.30]	1.53 [1.18—1.98]
No depression or anxiety ^d,g^	1329 (86.6)	1386 (92.2)	reference	reference	reference

^a^Due to matching with replacement, 33 double-used control patients were weighted as 0.5, all other patients were given a weight of 1.0 in all analyses.

^b^If a patient had both a disorder and a symptom code during follow-up, one occurrence of depression or anxiety was coded.

^c^In Model 1, univariate logistic regression analyses were performed. In Model 2, an ICPC above 60 was added as a covariate. In Model 3, ICPC above 60 was added as a covariate and only depression and anxiety that occurred six months or more after the index date were included to account for the possibility of reverse causality. If multiple symptoms and diagnosis codes were present during follow-up, the earliest code date was used to classify if depression or anxiety occurred after six months.

^d^Depression coded as the presence of either ICPC code P03, P76 or P76.02 in the medical record.

^e^Disorder coded as the presence of ICPC code P76 or P76.02 for depression and P74, P74.01 or P74.02 for anxiety in the medical record.

^f^Symptoms coded as the presence of P03 for depression and ICPC code P01 for anxiety in the medical record.

^g^Anxiety coded as presence of either ICPC code P01, P74, P74.01 or P74.02 in the medical record.

## Discussion

Patients with insomnia had a 1.5-fold increased risk to develop depression or anxiety in a five-year follow-up compared to healthy controls. For depression, this risk was increased 2.0-fold, and the risk for anxiety was increased 1.2-fold, although the latter OR had a broad 95% CI that reached lower than one. Including somatic comorbidity and accounting for possible reverse causality did not impact these results substantially.

The present study emphasizes the importance of asking about depression and anxiety during and following an insomnia consultation in general practice. In addition, treating insomnia accurately in this setting might reduce depression and anxiety incidences. Since CBT-I not only effectively treats insomnia but also reduces depressive symptoms in individuals with insomnia [20], CBT-I presumably reduces the risk for subsequent clinically significant depression. Although the effectiveness for reducing anxiety symptoms has not been well-established, an initial study illustrated the value of CBT-I in the prevention of anxiety disorders, including a decrease in anxiety symptoms in patients with insomnia [25]. This suggests CBT-I could be useful in this context. While enhancing implementation of CBT-I would be ideal, CBT-I interventions tailored to the primary care setting are not widely available, and sleep medication remains the most commonly provided treatment [17].

For the risk of depression, our results are comparable to the results of recent meta-analyses (OR = 2.10–2.83) [15 , 16]. For the risk of anxiety, the risk is less pronounced than the risk presented in the meta-analysis (OR = 3.23) [16]. The difference between depression and anxiety risks might be attributed to more accurate registration of depression in medical records than of anxiety. That is, while 26% and 7% of patients with an actual depressive disorder have a documented ICPC code for depressive disorder and depressive symptoms, the coverage of anxiety registration is considerably lower, ranging from 9 to 16% [6, 7]. These low registration rates may, together with the inclusion of patients without previous mental disorders, also contribute to the relatively low five-year cumulative incidences of depression and anxiety found in the present study, compared to the annual incidences of 1.7% for mood disorders and anxiety disorders each found in the NEMESIS-2 trial [26].

Differences in registration of anxiety relative to depression in primary care might also explain the differential outcomes of the sensitivity analyses. While in both conditions disorders used to be registered more often than symptoms, this pattern has reversed since the turn of the century [8, 9]. Although the reverse happened for both conditions, it seems to be more evident for anxiety, since more anxiety than depression symptoms have been reported in recent years, while for depression and anxiety disorder incidences stayed similar [5]. A recent qualitative study found GPs are reluctant to diagnose patients with anxiety disorders, because of stigmatisation and time restraints [27], offering a potential explanation for the observed trend towards relative under-registration of anxiety disorders.

Sleep influences emotion regulation on all levels [28]. This is supported by imaging studies that found that sleep deprivation increases amygdala reactivity and decreases prefrontal cortex-amygdala connectivity [29, 30]. Poor sleep furthermore impairs emotional processing [31], induces chronic inflammation [32], and increases neuro-endocrine stress responsiveness [33], potentially leading to the increased risk of patients with insomnia developing depression and anxiety.

A major strength of the present study is that the insomnia, depression, and anxiety diagnoses were retrieved from routine healthcare data, instead of self-reported questionnaires. This real-world data includes patients from different ethnicities and socio-economic backgrounds, acquiring a level of diversity not often achieved in traditional cohorts. By using primary care data, the present study shows that even when patients sought help for insomnia and GPs have probably provided treatment, the risk for depression and anxiety persists. Since help-seekers differ from non-help-seekers in aspects such as symptom severity and worrying [34, 35], the reported findings provide evidence that applies to the primary care setting. Including a measure for somatic comorbidity as a covariate in the analyses enhanced the applicability of the results further. Another strength comprises the consideration of both symptoms and disorders in the primary outcome of depression and anxiety, to account for variability in the registration of different mental disorders, in general, and between GPs. In addition, the present study accounted for the possibility of reverse causality and showed that this did not have a considerable effect on the risk.

The study also has some limitations. First, the fact that the diagnoses of insomnia, depression and anxiety are made by GPs is a strength, but also a weakness. Even though the diagnosis and symptom codes are generally based on established diagnostic criteria, this could not be verified by a standardized diagnostic instrument, like the Composite International Diagnostic Interview (CIDI) [36]. Second, not considering off-label medication prescriptions, such as low-dose quetiapine and mirtazapine, as medication for insomnia, may have led to missing patients with actual insomnia. However, due to their off-label use and alternative indications, including these medications would probably compromise the accuracy of patients with actual insomnia substantially. Third, patients with missing data were excluded, possibly resulting in selection bias. Because some general practices outsource financial tasks, not all registration fees of participating general practices are documented in the ELAN database. Since these registration fees are expected to be missing completely at random, this could compromise statistical power but would not introduce bias or affect the generalisability of the results. Another limitation is that patients with prior mental or social problems and psychoactive medication codes were excluded, resulting in a relatively resilient sample. This inclusion bias could have underestimated the risk of insomnia for subsequent depression and anxiety. Finally, patients with insomnia could have been predisposed to the development of depression and anxiety because of shared vulnerability factors [11] that could not be identified in the present study. While insomnia is an established risk factor with pathophysiological evidence supporting the causal relationship, it cannot be ruled out that insomnia may also represent a prodromal or an early symptom of depression and anxiety.

Using routinely collected primary care data, the present study reveals that patients with an insomnia diagnosis in primary care have an increased risk for receiving a subsequent diagnosis for depression and anxiety, despite the provision of usual care. Implementing prevention strategies that increase vigilance and improve insomnia treatment in primary care could reduce incidences of depression and anxiety on a population level. Future research is ongoing on how the management of insomnia in primary care can be improved.

## Ethical approval and consent to participate

The Medical Ethical Committee Leiden Den Haag Delft (MEC-LDD) issued a waiver of permission for this study (G20.197). Research with routine health care data from the Extramural LUMC Academic Network (ELAN) is observational, without an intervention, and enlisted patients are not individually approached for participation. In accordance with Dutch legislation, general practitioners informed individuals about the use of their pseudonymized data for research purposes, and individuals could withdraw via an informed opt-out procedure and informed consent from individuals in the study was waived and not obtained. For additional information in English, please refer to: https://english.ccmo.nl/investigators/legal-framework-for-medical-scientific-research/your-research-is-it-subject-to-the-wmo-or-not.

## Data Availability

The pseudonymized datasets used and/or analysed during the current study are not publicly available, but can be obtained after approval of a proposal via www.elanresearch.nl on reasonable request.
